# Multimodal neuroimaging computing: the workflows, methods, and platforms

**DOI:** 10.1007/s40708-015-0020-4

**Published:** 2015-09-04

**Authors:** Sidong Liu, Weidong Cai, Siqi Liu, Fan Zhang, Michael Fulham, Dagan Feng, Sonia Pujol, Ron Kikinis

**Affiliations:** 1 School of IT, The University of Sydney, Sydney, Australia; 2 Surgical Planning Laboratory, Harvard Medical School, Boston, USA; 3 Department of PET and Nuclear Medicine, Royal Prince Alfred Hospital, Sydney Medical School, The University of Sydney, Sydney, Australia; 4 Med-X Research Institute, Shanghai Jiao Tong University, Shanghai, China

**Keywords:** Multimodal, Neuroimaging, Medical image computing

## Abstract

The last two decades have witnessed the explosive growth in the development and use of noninvasive neuroimaging technologies that advance the research on human brain under normal and pathological conditions. Multimodal neuroimaging has become a major driver of current neuroimaging research due to the recognition of the clinical benefits of multimodal data, and the better access to hybrid devices. Multimodal neuroimaging computing is very challenging, and requires sophisticated computing to address the variations in spatiotemporal resolution and merge the biophysical/biochemical information. We review the current workflows and methods for multimodal neuroimaging computing, and also demonstrate how to conduct research using the established neuroimaging computing packages and platforms.

## Introduction

Neuroimaging has profoundly advanced neuroscience research and clinical care rapidly in the past two decades, prominently by magnetic resonance imaging (MRI), complemented positron emission tomography (PET), and electroencephalography (EEG)/magnetoencephalography (MEG). The art of neuroimaging today is shaped by three concurrent, interlinked technological developments [1]:

*Data Acquisition* The advances of imaging instrumentation have enabled digital image acquisition, as well as electronic data storage and communication systems, such as the picture archiving and communication system (PACS). These imaging systems, CT, MRI and PET showed obvious clinical benefits by providing high contrast tissue differentiation. The previous film-based reading was replaced by the electronic displays (axial, coronal and sagittal planes of the volume) without losing diagnostic quality.

*Medical Image Computing* The growth of neuroimaging has spurred a parallel development of neuroimaging computing methods and workflows, including bias correction, registration, segmentation, information extraction and visualization. We should note the difference between neuroimaging and neuroimaging computing. Neuroimaging focuses on the image acquisition, capturing the snapshot of the brain; whereas neuroimaging computing focuses on the computational analysis of the brain images, extracting and enhancing the information of relevance to best describe the brain anatomy and function.

*Package and Platform Development* To fit into research and clinical timelines and facilitate translational medicine, the neuroimaging computing methods and workflows are often integrated into software packages. Many such packages were added to imaging systems by the major vendors of medical imaging equipment and many specialized companies. However, a greater number of neuroimaging computing packages and platforms are free and open-source, designed and supported by the medical imaging research groups and communities.

Multimodal neuroimaging, i.e., the simultaneous imaging measurement (EEG/fMRI [2], PET/CT [3]) or summation of separate measurement (PET and sMRI [4], sMRI and dMRI [5], fMRI and dMRI [6]), has become an emerging research area due to better access to imaging devices, especially the hybrid systems, such as PET/CT [7, 8] and PET/MR [9]. The recent advances in neuroimaging computing methods also enabled joint analysis of the multimodal data. The free and open-source software (FOSS) packages and platforms for neuroimaging computing further facilitate the translation of the multimodal neuroimaging research from the lab to better clinical care.

Multimodal neuroimaging advances the neuroscience research by overcoming the limits of individual imaging modalities and by identifying the associations of findings from different imaging sources. Multimodal neuroimaging has been used to investigate a multitude of populations and disorders, such as Alzheimer’s disease (AD) [4, 10–12], schizophrenia [13–16], epilepsy [3, 17–19], obsessive-compulsive disorder (OCD) [20–22], bipolar disorder [23, 24], attention-deficit hyperactivity disorder (ADHD) [25–27], Autism spectrum disorder (ASD) [28–30], traumatic brain injury (TBI) [31–34], stroke [35, 36], multiple sclerosis (MS) [37–39], and brain tumors [9, 40–42]. We have recently reviewed advances in neuroimaging technologies and the applications of multimodal neuroimaging in these neuropsychiatric disorders [43]. Multimodal neuroimaging has also been used in many non-clinical applications, such as building brain machine interface (BMI) [44], tracing neural activity pathways [45] and mapping mind and behavior to brain anatomy [46–48].

Multimodal neuroimaging computing is a very challenging task due to large inter-modality variations in spatiotemporal resolution, and biophysical/biochemical mechanism. Compared to single imaging modality computing, it requires more sophisticated bias correction, co-registration, segmentation, feature extraction, pattern analysis, and visualization. Various methods for neuroimaging analysis have been proposed, and many have been integrated into the task-oriented packages or integrated platforms.

**Fig. 1:**
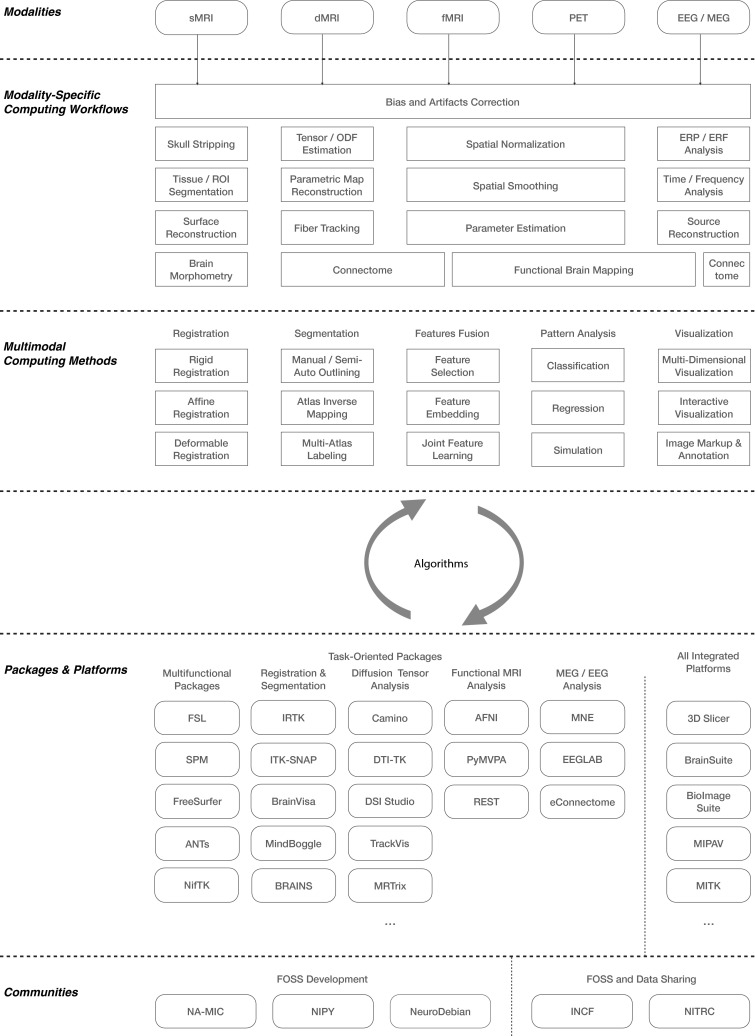
Overview of the current status and major components of multimodal neuroimaging computing, including neuroimaging modalities, modality-specific computing workflows, multimodal computing methods, algorithms, task-oriented packages, all-integrated platforms, and neuroimaging research communities

In this paper, we review the state-of-the-art methods and workflows for both modality-specific neuroimaging computing and multimodal neuroimaging computing, and demonstrate how to conduct multimodal neuroimaging research using the established packages and platforms. Fig. 1 provides an overview of the current status and illustrates the major components of neuroimaging computing, including neuroimaging modalities, modality-specific computing workflows (a series of tasks), multimodal computing methods, algorithms, packages, platforms and communities. MRI, PET, EEG/MEG and their computing workflows and methods are discussed in this review. A neuroimaging computing task in an analysis workflow may be fulfilled by multiple algorithms, and the most widely used algorithms, e.g., voxel-based morphometry (VBM) [49], are often integrated into software packages, e.g., Statistical Parametric Mapping (SPM)^1^, FMRIB Software Library (FSL)^2^, and Neurostat^3^. The new imaging tasks also demand the refinement of existing algorithms and development of new algorithms. Similar algorithms are often developed independently in different labs, sometimes with little awareness of existing packages/platforms.

This paper is organized as follows. In Sect. 2, we elaborate the computing workflows, which consist of a number of specific tasks, for individual modalities. In Sect. 3, we review the major multimodal neuroimaging computing methods, i.e., registration, segmentation, feature integration, pattern analysis and visualization. In Sect. 4, we introduce the task-oriented packages and platforms for the tasks mentioned in previous sections. We focus on the free and open source software (FOSS) in this review, since they could help to better realize the quickly evolved methods and workflows than their commercial counterparts, and thus accelerate translational medicine. For the sake of clarity and precision, the algorithms, packages and platforms are not described in detail, but we refer the interested readers to more specific papers instead. In Sect. 5, we give one example of brain tumor surgical planning using the established packages and platforms. Lastly, we outline the future directions of multimodal computing in Sect. 6.

## Modality-specific neuroimaging computing workflows

### Bias and artifacts correction

Different neuroimaging techniques have different spatiotemporal resolutions, and biophysical/biochemical mechanisms, and thereby require different computing workflows; yet a common step of all workflows is the correction of bias and artifacts in neuroimaging data. The main goal of this task is to remove the data components that contaminate the signals. Tustison recently provided a set of guidelines for managing the instrumental bias when designing and reporting neuroimaging studies [50].

Bias and artifacts in neuroimaging signals may result from imaging systems, environment, and body motion. Many biases and artifacts are induced by the imaging systems, e.g. inhomogeneous radio frequency (RF) coils in MRI, contrast agents in PET/CT, broken or saturated sensors in EEG/MEG system. Environment-related artifacts, arising from generators of magnetic fields outside the human body such as magnetic noise from power lines and other environmental noise sources, such as elevators, air conditioners, nearby traffic, mechanical vibrations transmitted to shielded room, bed vibration, and pulsation [51]. Motion-related artifacts are caused by eye movements, head movements, cardiac and muscular activity, and respiratory motion. The motion of magnetic implements, such as pacemakers and implantable defibrillators [52] may also give rise to artifacts, and may cause danger to patients in strong magnetic field, although there are new MRI compatible pacemakers/defibrillators that have been introduced [53].

The bias and artifacts in MRI are mainly system-related, e.g., RF inhomogeneity causing slice and volume intensity inconsistency. The nonparametric nonuniformity normalization (N3) algorithm and its variant based on Insight Toolkit [54, 55] (N4ITK) [56] are the *de facto* standard in this area. The acquisition protocols for dMRI are inherently complex, which require fast gradient switching in Echo-Planar Imaging (EPI) and longer scanning time. dMRI is prone to many other types of artifacts, such as eddy current, motion artifacts and gradient-wise inconsistencies [57]. Tortoise [58] and FSL diffusion toolbox (FDT) [59] are popular choices for eddy current correction and motion correction in dMRI data, and the recently proposed DTIPrep [60] offers a thorough solution for all known data quality problems of dMRI. Motion is a serious issue in fMRI, and may lead to voxel displacements in serial fMRI volumes and between slices. Therefore, serial realignment and slice timing correction is required to eliminate the effects of head motion during the scanning session. Linear transformation is usually sufficient for serial alignment, whereas a non-linear auto-regression model is often used for slice timing correction [61]. These two types of correction are commonly performed using SPM and FSL. Dedicated PET scanners have been replaced by the hybrid PET/CT systems [62]. The most commonly seen artifacts on PET/CT are mismatches between CT and PET images caused by body motion due to the long acquisition time of the scan. Metallic implants and contrast agents may also give rise to artifacts on PET/CT, usually leading to overestimation of PET attenuation coefficients and false-positive findings [63]. Knowledge and experience are needed to minimize these artifacts and, in that way, produce better-quality PET/CT images. EEG and MEG signals are often contaminated by all of the three types of artifacts, such as the system-related superconducting quantum interference device (SQUID) jumps, and the noise from the environment or body motion [51]. Visual checks and manual removal are usually required to exclude the artifacts. Another strategy uses signal-processing methods to reduce artifacts while preserving the signal. Linear transformation, e.g., principal component analysis (PCA) and independent component analysis (ICA) [64, 65], and regression, e.g., signal space projection (SSP) and signal space separation (SSS) [66, 67], are frequently applied to the raw EEG/MEG data.

### Structural MRI computing

The sMRI computing workflows usually involve skull striping, tissue and region of interest (ROI) segmentation, surface reconstruction [68], and can include brain morphometry analysis, such as the voxel-based morphometry (VBM)/tensor-based morphometry (TBM)/deformation-based morphometry (DBM) [49], and surface-based morphometry (SBM) [69] by comparing one group of subjects to another or tracking the changes over a sequence of observations for the same subject. FreeSurfer [70] is a well-established tool for brain tissue segmentation and surface reconstruction. When registered into a standard brain space, e.g., the Talariach coordinates [71] and MNI coordinates [72], and labeled with different regions of interest (ROIs) using brain templates, e.g., ICBM template [73] and the AAL template [74], the sMRI datasets can further be analyzed at the ROI level. Various techniques have been investigated to quantitatively analyze the morphological changes in cortex, e.g., grey matter density [49], cortical folding [75], curvedness and shape index [76, 77], cortical thickness [69], and surface area [78, 79], local gyrification index (LGI) [75], and many other shape [78, 80] or texture features [81–83]. Mangin et al. [84] provided an extensive review on the popular morphological features, and Winkler et al. [85] demonstrated how to use these features in imaging genetics.

### Diffusion MRI computing

The dMRI computing workflow consists of four major steps. The first step is to estimate the principle directions of the tensor or the orientation distribution function (ODF) in each voxel, which are used to quantitatively analyze the local white matter morphometry and probe the white matter fiber tracts in the following steps. Advanced fiber orientation estimation methods include the ball and stick mixture models [59], the constrained spherical deconvolution (CSD) [86], the q-ball imaging (QBI) [87], diffusion spectral imaging (DSI) [88], the generalized q-sampling imaging (GQI) [89], and the QBI with Funk Radon and Cosine Transform (FRACT) [90]. Wilkins et al. have provided a detailed comparison of these models [91]. In the second step, various parametric maps based on the tensors/ODFs, i.e., fractional anisotropy (FA), mean diffusivity (MD), radial diffusivity (RD), and axial diffusivity (AXD) maps [92], reveal the focal morphometry of the white matter [93]. The third step is to apply the fiber tracking algorithms [94] to construct 3D models of the white matter tracts, referred to as tractography. Tractography further enables the quantitative analysis of fiber tract morphometry i.e., orientation and dispersion [95], and the analysis of connectome, i.e., connectivity networks of populations of neurons [96]. Brain parcellation and fiber clustering are two major approaches that can separate the neurons into different groups/ROIs, and construct the connectome [97]. Jones et al. [98] recently provided a set of guidelines which define the good practice in dMRI computing.

### Functional MRI computing

After bias and artifacts correction in fMRI, a mean image of the series, or a co-registered anatomical image, e.g., sMRI, is used to estimate some registration coefficients that map it onto a template, followed by spatial smoothing and parameter estimation. Friston [99] gave an introduction to these procedures. When the brain is performing a task, cerebral blood flow (CBF) usually changes as neurons work to complete the task. The primary use of task-evoked fMRI is to identify the correlation between brain activation pattern and brain functions, such as perception, language, memory, emotion and thought [100, 101]. Many models and methods have been suggested to detect patterns of brain activation, and some of them have been integrated into the software packages, such as the general linear model (GLM) in the SPM and FSL packages, and independent component analysis (ICA)/canonical correlation analysis (CCA) in AFNI package^4^. When brain is at resting state, fMRI is used to detect the spontaneous activation pattern in the absence of an explicit task or stimuli [102]. Resting-state fMRI enables us to deduce the functional connectivity between dispersed brain regions, which form functional brain networks, or resting state networks (RSNs). The Default Mode Network (DMN) is a functional network of several brain regions that show increased activity at rest and decreased activity when performing a task [103]. DMN has been widely used as a measure to compare individual differences in behavior, genetics and neuropathologies, although its use as a biomarker is controversial [104, 105]. Rubinov [106] provided a review of the connectivity measures.

### PET computing

The computing of PET also requires spatial normalization and smoothing, and parameter estimation, similar to fMRI. SPM and Neurostat packages are available for voxel-by-voxel PET analysis. PET functional features are generally pertaining to the radioactive tracers, reflecting particular biochemical process. 2-[^18^F fluoro-2-deoxy-D-glucose (FDG) is the most widely used tracer to depict glucose metabolism. Several amyloid-binding compounds, $^{18}$F-BAY94-9172, $^{11}$C-SB-13, $^{11}$C-BF-227, $^{18}$F-AV-45 and $^{11}$*C-Pittsburgh compound B* ($^{11}$C-PiB), have been reported as tracers for imaging amyloid plaques in AD. A number of extensive surveys have been conducted on these amyloid radioactive tracers [107–110]. A variety of static and kinetic parameters can be extracted from the PET data, i.e. the standard uptake value (SUV) [111, 112], cerebral metabolic rate of glucose consumption (CMRGlc) [81, 113], mean index [114], z-scores [115], hypo-metabolic convergence index (HCI) and amyloid convergence index (ACI) [116], tissue time activity curve (TTAC) [117], and difference-of-Gaussian (DoG) parametric maps [118].

### EEG and MEG computing

In EEG and MEG there are usually four components after removing the artifacts or unwanted data components that contaminate the signals. The analysis of event-related potentials (ERP) in EEG or event-related fields (ERF) in MEG aims to analyze brain responses that are evoked by a stimulus or an action, followed by spectral analysis, which transforms the signals into time-frequency domain. The aim of source reconstruction is to localize the neural sources underlying the signals measured at the sensor level. MRI is usually used to provide anatomical reference for source reconstruction. The aim of connectome analysis is to investigate the causality of brain activities and connectivity of brain networks by exploring information flow and interaction between brain regions. Gross et al. provided basic guidelines on EEG and MEG in research [51]. MNE^5^, EEGLAB^6^ and eConnectome^7^ are the most widely used software packages specifically designed for EEG and MEG computing.

## Multimodal neuroimaging computing methods

### Registration

Registration is the most commonly used technique in a neuroimaging study, and it finds the spatial relationship between two or more images, e.g., multimodal neuroimaging data alignment, serial alignment, and atlas mapping. A registration method can be defined in five aspects, i.e., a cost function for evaluating the similarity between images, a transformation model to determine the degree-of-freedom (DOF) of the deformation, an optimization method for minimizing the cost function, a sampling and interpolation strategy for computation of the cost function, and a multi-resolution scheme for controlling the coarseness of the deformation [119].

Registration methods can be roughly classified into three categories according to the DOF of their transformation models. Rigid registration has a DOF of 6 and allows for global translations and rotations. Affine registration, i.e., linear registration, allows for translation, rotation, scaling and skew of the images. Rigid and affine registration methods are usually sufficient for registering the multimodal datasets of same subject. However, deformable registration, which supports local deformations, is frequently needed to register images with large differences, e.g., registering an image to a template, or registering pre- and post-contrast images of the same subject. Deformable registration always requires rigid or affine registration to obtain a rough initial alignment. In many multimodal studies, a combination of these registration methods were used. For example, we recently jointly analyzed the ADNI FDG-PET and T1-weighted MRI datasets to classify AD and mild cognitive impairment (MCI) patients [79]. The PET images were aligned to MRI using an affine registration method (FSL FLIRT) [120]. The MRI datasets were registered to the MNI template using a deformable method (IRTK) [121], and the output registration coefficients by IRTK were applied to register the PET images to the same template. There are many other widely used registration algorithms, such as B-Spline registration [119, 122], Demons [123], and SyN [124], and ITK [54] registration framework is a standard-bearer for all of these popular registration methods.

### Segmentation

Segmentation is also referred to as brain parcellation or labeling. The brain can be segmented at different levels, i.e., tissues (grey matter, white matter, cerebrospinal fluid), cortical regions, and sub-cortical regions. The segmentation methods can be classified into three categories [125]. The first category is manual and semi-automatic methods, which require manually outlining the brain regions according to a protocol [126, 127] or labeling the landmarks or seed points [128, 129]. These methods are labour-intensive and prone to intra- and inter-operator variation.

The second category is the atlas inverse mapping methods, which can inversely map a labeled atlas, e.g., the standard ICBM and AAL template, or user-defined image, to the original image space. Yao et al. recently provided a review of popular brain atlases [130]. Atlas inverse mapping is simple, but its performance heavily depends on the selected atlas and mapping method.

A more robust but complex solution is multi-atlas labeling, including the multi-atlas propagation with enhanced registration (MAPER) [131] and its variants [132, 133]. These methods carry out whole-brain segmentation in the original image space by fusing multiple labeling results derived from the multiple atlases. Multi-atlas labeling is computationally expensive, but the performance is comparable to manual labeling [125]. FSL FAST^8^ and NifSeg^9^ are widely used for brain tissue segmentation. IRTK, Advanced Normalization Tools (ANTs)^10^ and NifReg^11^ are commonly used in multi-atlas labeling as the normalization tools.

### Feature fusion

Various features can be extracted from the neuroimaging data, as described in Sect. 2. Feature fusion is needed to jointly analyze the features from multimodal data. A straightforward solution is to concatenate input multi-view features into a high-dimensional vector, and then apply feature selection methods, such as t-test [134], ANOVA [118], Elastic Net [10, 135], lasso [136] or a combination of these methods [137, 138], to reduce the ’curse of dimensionality’.

These methods show promising results. However, the inter-subject variations cannot be eliminated using the concatenation methods because the inter-subject distances measured by different features may have different scales and variations. With a focus on the subjects, the feature embedding methods, such as multi-view spectral embedding (MSE) [139] and multi-view local linear embedding (MLLE) [140], have been used to explore the geometric structures of local patches in multiple feature spaces and align the local patches in a unified feature space with maximum preservation of the geometric relationships.

In addition, machine learning, especially deep learning, is increasingly used to extract high-level features from neuroimaging data. The advantage of learning-based features is they do not depend on prior knowledge of the disorder or imaging characteristics as the hand-engineered features. They are also essentially suitable for multimodal feature learning, and could expect better performance with larger datasets. However, learning-based features heavily depend on the training datasets [141]. Recently, Suk et al. [142] proposed a feature representation learning framework for multimodal neuroimaging data. One stacked auto-encoder (SAE) was trained for each modality, then the learnt high-level features were further fused with a multi-kernel support vector machine (MK-SVM). They further proposed another deep learning framework based on the deep Boltzmann machine (DBM) and trained it using the 3D patches extracted from the multimodal data [143].

### Pattern analysis

Pattern analysis aims to deduce the patterns of disease pathologies, sensorimotor or cognitive functions in the brain and identify the associated regionally specific effects. A substantial proportion of pattern analysis methods focused on classification of different groups of subjects, e.g., distinguishing AD patients from normal controls [10, 138]. Hinrichs et al. [144, 145] and Zhang et al. [4] recently proposed the multi-kernel support vector machine (MK-SVM) algorithm, which is based on multi-kernel learning and extends the kernel tricks in SVM to the multiple feature spaces. We previously proposed a multifold Bayesian kernelization (MBK) model [79] to transfer the features into diagnosis probability distribution functions (PDFs), and then merge the PDFs instead of the feature spaces.

Regression-based pattern analysis is often used to identify the biomarkers of a group of subjects and probe the boundaries between different groups. The multimodal biomarkers can be based on the voxel features, ROI features and other features, as described in Sect. 2. Regression, such as Softmax regression [10], Elastic Net [135], and lasso [136] can be combined with feature learning in a unified framework.

Recently, the pattern analysis methods have been extended to simulation of future brain development based on the previous states of the brain and comparison to other brains. The basic assumption is that brains with similar cross-sectional and longitudinal deformations would have similar follow-up development [146, 147]. When the population is sufficiently large to include a majority of neurodegenerative changes, the simulated results are more accurate.

### Visualization

The neuroimaging data are mainly 2D and 3D, thus can be visualized in multi-dimensional spaces with 2D and 3D viewers. Multimodal data in 2D space are usually displayed with three layers, including background, foreground and label maps. The 3D viewer enables visualization of volume data, such as volume renderings, triangulated surface models and fiber tracts. Basic image visualization functions, such as look up tables, zoom, window / level, pan, multi-planar reformat, crosshairs, and synchronous pan / scroll for linked viewers, have been implemented in most visualization platforms, such as Slicer^12^ and BioImage Suite^13^. These platforms also can accommodate visualization of high-dimensional data, e.g., tensors and vector fields.

Image markup refers to the graphical elements overlay, such as fiducials (points), rulers, bounding boxes, and label maps. Image annotation refers to the text-based information [148]. Both image markups and annotations are used to describe the meta information of the images, and annotations can be associated with markup elements as free text.

Another important use of the image markups is interactive visualization. The aforementioned platforms also provide a graphical user interface to interact with the data. For example, the volume rendering module of Slicer allows the users to define a bounding box and visualize the content in the bounding box only. Another module, tractography interactive seeding, is designed for interactive seeding of DTI fiber tracts passing through a list of fiducials or vertices of a 3D model. Slicer also allows the configuration of the layouts and manipulation of content in the viewers to suit a specific use case.

## FOSS packages and platforms

### Task-oriented packages

The FOSS packages for neuroimaging computing are usually initially designed for a single task, such as registration and segmentation, and some of them then are extended to related tasks and become multifunctional packages. A number of the most widely used FOSS packages are listed in Fig. 1—packages and platforms.

Popular multifunctional packages include FreeSurfer, FSL, SPM, ANTs and NifTK. They cover similar aspects of functionality, but all have particular strengths. FreeSurfer and FSL provide a comprehensive solution of analysis tools for fMRI, sMRI and dMRI data. SPM is designed for the analysis of fMRI, PET, SPECT, EEG and MEG. The recently developed ANTs and NifTK are useful for managing, interpreting and visualizing multimodal data, and represent the state-of-the-art in medical image registration and segmentation. Tustison et al. [149] recently compared ANTs and FreeSurfer in a large-scale evaluation of cortical thickness measurements. Other packages may focus on a specific task or a set of related tasks. IRTK^14^, BRAINs [150], BrainVisa^15^, ITK-SNAP^16^ and MindBoggle^17^ are popular choices for registration and segmentation. In dMRI analysis, Camino^18^, DTI-TK^19^, DSI Studio^20^, TrackVis^21^ and MRTrix^22^ are most widely used packages. Soares et al. [151] recently conducted a thorough evaluation of these packages and the other dMRI computing packages in published studies. In functional neuroimaging computing, AFNI, PyMVPA^23^ and REST^24^ are widely used for fMRI analysis, whereas MNE, EEGLAB, eConnectome for EEG/MEG analysis.

### All integrated platforms

For clinical applications, the medical image computing and visualization functions are part of the operation system and must meet the same standards of reliability, robustness, and simplicity of operation as the core imaging equipment. This is usually accomplished using software platforms added onto imaging system by the major vendors of medical image equipment and many specialized companies. Examples include Advantage Windows (General Electric), Syngo Via (Siemens), Vital Image Vitrea (Toshiba), Visage Amira (Visage Imaging), PMOD (PMOD Technologies Ltd.), Definiens (Definiens Inc.), and MimVista (MIM Software Inc.). These packages provide users with a set of analysis tools, compatibility with PACS and customer support. Such clinically oriented systems are not always affordable for academic researchers. Commercial solutions are typically not extensible by the end user, nor oriented towards prototyping of new tools, and may require specialized hardware, thereby limiting their applicability in projects that involve the development of new image computing methods.

As opposed to the commercial platforms, FOSS platforms are meant to provide a research platform that is freely available and does not require specialized hardware. A key step in the evolution of today’s flexible and sophisticated capabilities for image-data-based research medicine was the creation of the 3D Slicer, which is based on a modular architecture [1, 152]. 3D Slicer has become a successful and long-lived platform for the effective use of volumetric images in clinical research and procedure development. There are a number of platforms which aim to cover similar aspects of functionality, e.g., BioImage Suite, BrainSuite^25^, MIPAV^26^ and MITK^27^.

Some of the libraries contributing to the foundation of Slicer were designed in close collaboration and often share the same developer community. These libraries, including CMake, ITK, VTK and CTK, are distributed as part of the National Alliance for Medical Image Computing (NA-MIC) Kit [153], which are actively supported by the NA-MIC research community^28^. Many popular packages, e.g., ANTs, MindBoggle, ITK-SNAP, DTIPrep, and MITK are also based on the NA-MIC Kit. NIPY^29^ and NeuroDebian^30^ are another two major research communities for neuroimaging research and platform development. To promote open science, neuroimaging tools and resources are always shared to other community members, usually through the INCF^31^ and NITRC^32^ forums.

**Fig. 2:**
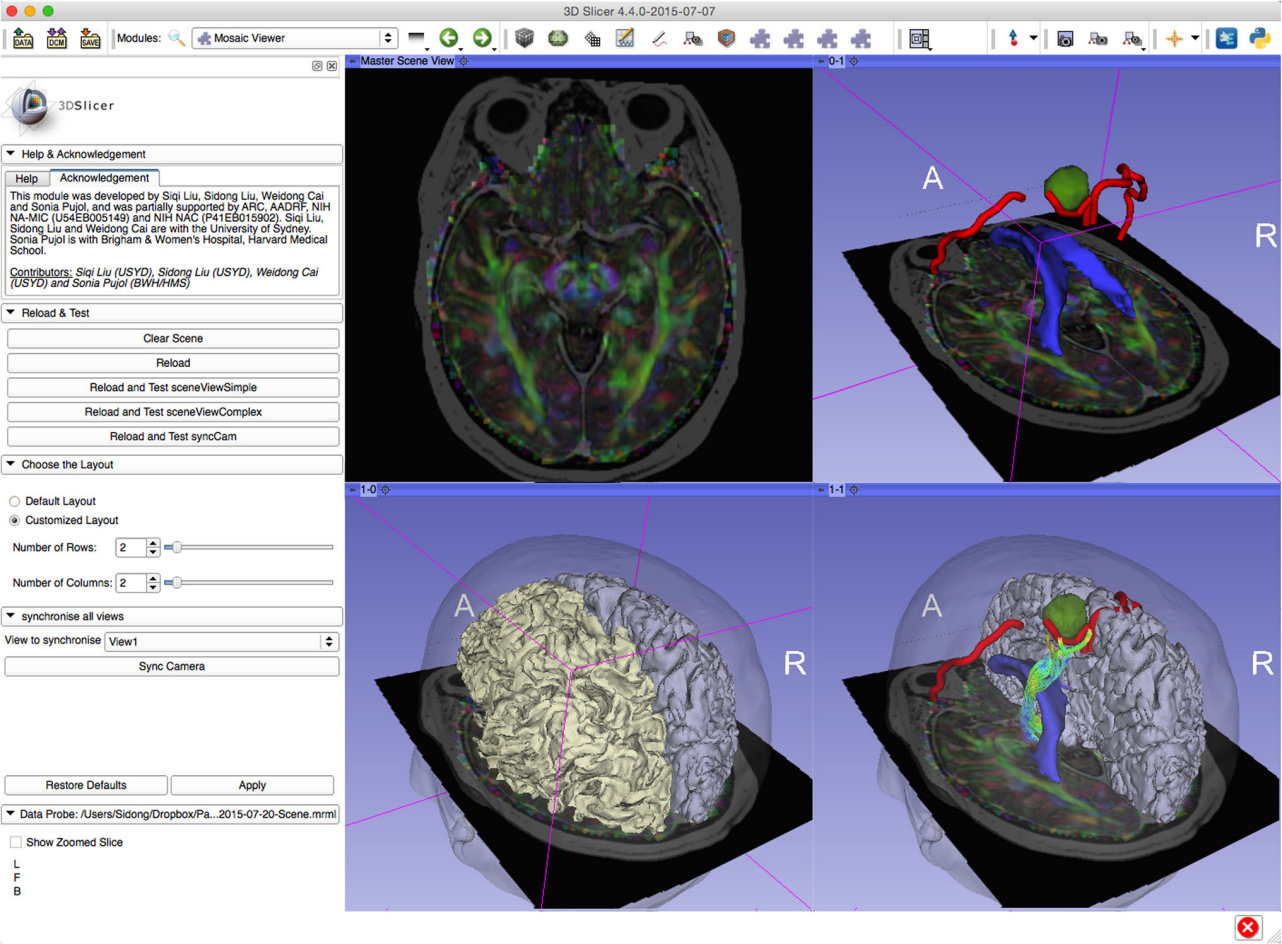
Experimental visualization of brain tumor case of DTI Challenge 2015 using 3D Slicer. The *panel on the left* shows the GUI of the Slicer Mosaic Viewer module previously developed by us. The *right side* shows the four data viewers, each visualizing a specific step in the surgical planning workflows. The *up left viewer* shows the registered T1 that overlaid on the DTI volume. The *up right viewer* shows the segmented tumor (*green*), ventricle (*blue*), and motor cortex (*red*) surfaces. The *bottom left viewer* shows the reconstructed pial surface of the right hemisphere and white matter surface of the left hemisphere. The *bottom right viewer* interactively visualizes the peritumoral fiber tracts as the user moves the fiducial. (Color figure online)

## Example: surgical planning for brain tumor resection

Tractography derived from dMRI has great potential to help neurosurgeons determine tumor resection boundaries in functional areas involving eloquent white matter fibers. The MICCAI DTI Challenge^33^ is dedicated to comparing different fiber-tracking algorithms in reconstruction of white matter tracts, such as peritumoral tracts and cerebrospinal tract (CST). In this section, we present an example of pre-operative planning for brain tumor resection using the sMRI and dMRI data. The original data consist of a DWI volume and two structure scans of a patient with meningioma. The DWI scan was acquired with a spin-echo EPI sequence with the following parameters: voxel size 2.2 $\times$ 2.2 $\times$ 2.2 mm, FOV 220 mm, 58 slices, b-value 1000 s/mm$^{2}$, 30 diffusion-weighted volumes and 1 baseline volume. The T1 original was acquired using a Ax 3D T1 MPRAGE sequence. The T2 original was acquired using a Ax 3D SPACE sequence.

The original data were computed in four steps, as illustrated in Fig. 2. For dMRI-specific computing, the tensors were estimated using a weighted least square (WLS) algorithm, and the output is a DTI volume. We then registered the T1 and T2 MRI volumes to baseline volume using the affine registration algorithm. The registered T1 and T2 volumes were used as the anatomical references. For sMRI-specific computing, tumor, ventricle and motor cortex were manually seeded and semi-automatically labeled in the baseline volume. The label map of the tumor and ventricle were than used to generate the 3D surface model using the Model Maker module in Slicer. The head surface, pial surface and white matter surfaces for both hemisphere were reconstructed using the Morphologist 2013 pipeline in BrainVisa [68]. For multimodal computing, the white matter tracts were visualized using the Slicer Tractography Interactive Seeding module, which allows users to mark the image with fiducials, and then move it around the tumor to visualize the peritumoral fiber tracts.

## Future directions

The neuroimaging techniques will keep advancing rapidly, towards higher spatial/temporal/angular resolutions, shorter scanning time, and greater image contrast. In particular, the advances in the hybrid imaging scanners, e.g., PET/CT and PET/MRI, will enter more clinics and laboratories, lowering the cost for data acquisition and enabling more interesting discoveries in a greater multitude of populations and disorders.

The continued growth in the complexity and dimensionality of neuroimaging data will spur the parallel advances of computational models and methods to accommodate such complex data. Such models and methods need to keep increasing the grade of automation, accuracy, reproducibility and robustness, and eventually need to be integrated into the clinical workflows to facilitate clinical testing of the new neuroimaging biomarkers.

The multidisciplinary nature of neuroimaging computing will keep bringing together clinicians, biologists, computer scientists, engineers, physicists, and other researchers who are contributing to, and need to keep abreast of, advances in the neurotechnologies and applications. New methods and models will be developed by the collaboration of different groups or individuals, with rapid iterations. Therefore, future packages and platforms need to respond more quickly to the updates, without compromising the functionality, extensibility and portability. This might cause difficulties in the maintenance of large packages and platforms, but will encourage the researchers to provide smarter solutions, e.g., providing an online version to make the whole process of developing, sharing and updating much quicker for both developers and users.

## Footnotes

1 www.fil.ion.ucl.ac.uk/spm.

2 www.fmrib.ox.ac.uk/fsl.

3 http://128.208.140.75/~Download/.

4 http://afni.nimh.nih.gov/.

5 http://martinos.org/mne/.

6 http://sccn.ucsd.edu/eeglab/.

7 http://econnectome.umn.edu/.

8 http://fsl.fmrib.ox.ac.uk/fsl/fslwiki/FAST.

9 http://cmictig.cs.ucl.ac.uk/wiki/index.php/NiftySeg.

10 http://stnava.github.io/ANTs/.

11 http://cmictig.cs.ucl.ac.uk/wiki/index.php/NiftyReg.

12 http://www.slicer.org/.

13 http://bioimagesuite.yale.edu/.

14 https://www.doc.ic.ac.uk/~dr/software/.

15 http://brainvisa.info/.

16 http://www.itksnap.org/.

17 http://www.mindboggle.info/.

18 http://cmic.cs.ucl.ac.uk/camino/.

19 http://dti-tk.sourceforge.net/.

20 http://dsi-studio.labsolver.org/.

21 http://trackvis.org/.

22 https://github.com/MRtrix3.

23 http://www.pymvpa.org/.

24 http://restfmri.net/.

25 http://brainsuite.org/.

26 http://mipav.cit.nih.gov/.

27 http://mitk.org/wiki/MITK.

28 http://www.na-mic.org/.

29 http://nipy.org/.

30 http://neuro.debian.net/.

31 http://www.incf.org/.

32 http://www.nitrc.org/.

33 http://projects.iq.harvard.edu/dtichallenge15/home.
